# ANNOUNCEMENT / NOUVELLE

**DOI:** 10.29173/jchla29838

**Published:** 2025-04-01

**Authors:** Jeff Mason, Patricia Foster

**Affiliations:** Co-Chairs, 2025 Conference Program Committee

*[Le français suit]*

**Join us in Vancouver from June 3 to 6, 2025 for our annual conference!**

We are delighted to invite you to the Canadian Health Libraries Association (CHLA) annual conference in vibrant Vancouver, British Columbia, from June 3 to 6, 2025. This year's theme, “Building Futures Together,” reflects our dedication to collaborative growth and innovation in health libraries.

The conference will feature esteemed keynote speakers, including a closing address by Dr. Devon Greyson, Assistant Professor at the University of British Columbia's School of Population and Public Health. Dr. Greyson's research focuses on public health communication and the use of information by the public, clinicians, and health systems.

The program will include a diverse array of sessions, workshops, and panel discussions contributed by health library professionals from across Canada and internationally.

The conference will be held at The Nest, University of British Columbia, a state-of-the-art facility located at 6133 University Blvd, Vancouver, BC. Join us for the Welcome Reception at The Nest on Tuesday, June 3, 2025, and take advantage of opportunities to build professional relationships and exchange ideas through a variety of social events, including informal dine-arounds, tours, and a banquet.

Early bird registration is open until April 30, 2025. We are committed to inclusivity and accessibility, offering reduced rates for library technicians, students, retirees, unemployed individuals, and equity-deserving groups. Members of the Medical Library Association are eligible for member rates.

Please visit the official conference website for the latest updates on the conference program, registration, and social events. We look forward to welcoming you to Vancouver in June 2025. Let's build the future of health librarianship together!

**Jeff Mason and Patricia Foster**

*Co-Chairs, 2025 Conference Program Committee*

**Joignez-vous à nous à Vancouver du 3 au 6 juin 2025 pour notre conférence annuelle!**

Nous avons le plaisir de vous inviter à la Conférence annuelle de l'Association des bibliothèques de la santé du Canada (ABSC) qui se tiendra dans la dynamique ville de Vancouver, en Colombie-Britannique, du 3 au 6 juin 2025. Le thème de cette année, « Construire des futurs ensemble », reflète notre dévouement à la croissance collaborative et à l'innovation dans les bibliothèques de la santé.

La conférence accueillera d'éminents conférenciers invités, dont Devon Greyson, professeur adjoint à la School of Population and Public Health de l'Université de Colombie-Britannique, qui prononcera le discours de clôture. Les recherches du Dr Greyson portent sur la communication en santé publique et sur l'utilisation de l'information par le public, les cliniciens et les systèmes de santé.

Le programme comprendra un large éventail de sessions, d'ateliers et de tables rondes animés par des professionnels des bibliothèques de la santé de partout au Canada et à l'international.

La conférence se tiendra à The Nest, University of British Columbia, une installation ultramoderne située au 6133 University Blvd, Vancouver, C.-B. Joignez-vous à nous pour la réception de bienvenue au Nest le mardi 3 juin 2025, et profitez des occasions de nouer des liens professionnels et d'échanger des idées à travers une variété d'événements sociaux, y compris des tournées gastronomiques, des visites guidées et un banquet.

L'inscription anticipée est ouverte jusqu'au 30 avril 2025. Nous nous engageons à favoriser l'inclusion et l'accessibilité en offrant des tarifs réduits pour les techniciens de bibliothèque, les étudiants, les retraités, les personnes sans emploi et les groupes méritant l’équité. Les membres de la Medical Library Association peuvent bénéficier des tarifs réservés aux membres.

Veuillez consulter le site officiel de la conférence pour les dernières mises à jour sur le programme de la conférence, les inscriptions et les activités sociales. Nous nous réjouissons de vous accueillir à Vancouver en juin 2025. Construisons ensemble l'avenir des bibliothèques de la santé!

**Jeff Mason et Patricia Foster**

*Coprésidents du comité de planification de la conférence 2025*

